# Haplotype analysis of DQ2.5 and DQ8 by simple nucleotide polymorphism technique (TAG-SNP) in type 1 diabetes and/or celiac disease patients

**DOI:** 10.1186/1758-5996-7-S1-A222

**Published:** 2015-11-11

**Authors:** Marília Dornelles Bastos, Thayne Woycinck Kowalski, Luiza Monteavaro Mariath, Márcia Khaled Puñales, Balduíno Tschiedel, Ana Carolina Moisés da Silva, Maria Eduarda Tavares, Ana Luiza Guedes Pires, Lavínia Schüler Faccini, Themis Reverbel da Silveira

**Affiliations:** 1 Universidade De Santa Cruz Do Sul-UNISC, Porto Alegre, Brazil

## Background

Celiac disease (CD) is a chronic and permanent enteropathy triggered by ingestion of gluten proteins, present in wheat, rye and barley, in genetically predisposed individuals, as type 1 diabetes mellitus (T1D) patients, who have a higher prevalence of the disease compared to the general population. Both diseases have similar autoimmune origin, being associated to the histocompatibility complex class II antigen (HLA), mainly DQ2.5 and/or DQ8 haplotypes, allowing estimating negative predictive value accurately. Nevertheless, these genetic tests are expensive, making its implementation a challenge in routine clinical practice.

## Objective

The aim of the study was the evaluation of the frequency of DQ2.5 and DQ8 haplotypes in T1D and CD patients, using a simple nucleotide polymorphism technique (Tag-SNP).

## Materials and methods

The study enrolled 365 individuals, being 296 with T1D (without CD=265 and with CD=31) and 69 with only CD. The HLA-DQA1* 0501 and DQB1* 0201alleles of DQ2.5 and HLA-DQB1*0302 allele of DQ8 were analyzed by HLA Tag SNP and its frequency compared between T1D and CD.

## Results

The presence of DQ2.5 alleles was found in 57.1% (169/296) among T1D, being significantly more frequent among T1D individuals with CD (without CD 54.3% and with CD 80.6%, p=0.006). There was no significant difference in the comparison among T1D with CD and CD individuals without diabetes (80.6% and 62.3%, p=0.054). The presence of the DQ8 allele was found 54.2% (160/295) among all T1D (without CD 51.6% and with CD 48.4%,p=0.569).The DQ8 allele was significantly higher among T1D with CD when compared to CD individuals without diabetes (72.6% and 29.5%, p<0.001).

## Conclusion

Our data evidenced a higher frequency of HLA-DQB1*0302 allele of DQ8 in T1D than CD individuals without diabetes, which did not exclude CD in this group of patients. However, the analysis of HLA-DQA1* 0501 and DQB1* 0201 alleles of DQ2.5 is useful in the evaluation of the risk of CD in predisposed individuals as T1D patients.

